# An investigation of movement dynamics and muscle activity during traditional and accentuated-eccentric squatting

**DOI:** 10.1371/journal.pone.0276096

**Published:** 2022-11-01

**Authors:** Richard Armstrong, Vasilios Baltzopoulos, Carl Langan-Evans, Dave Clark, Jonathan Jarvis, Claire Stewart, Thomas O’Brien

**Affiliations:** Research Institute for Sport and Exercise Science, Liverpool John Moores University, Liverpool, United Kingdom; University of Castilla-La Mancha, SPAIN

## Abstract

**Introduction:**

Accentuated-eccentric loading (AEL) takes advantage of the high force producing potential of eccentric muscle contractions, potentially maximising mechanical tension within the muscle. However, evidence is lacking on how AEL squatting may load the involved musculature, limiting scientifically justified programming recommendations. The purpose of this study was to investigate the effects of concentric and eccentric loads on joint loading and muscle activity of the lower limbs.

**Methods:**

Resistance trained males performed traditional squatting (20–100% of concentric one-repetition maximum [1RM]) and AEL squatting with eccentric loads (110–150% of 1RM) provided by a novel motorised isotonic resistance machine (Kineo). Kinetics and kinematics of the hip, knee, and ankle joints were collected, with electromyography from the gluteus maximus, vastus lateralis, biceps femoris, and gastrocnemius medialis. A secondary cohort underwent a kinematic and electromyography analysis of squatting technique to compare Kineo and back and front barbell squatting.

**Results:**

Knee joint peak eccentric moments occurred at 120% 1RM (*P* = 0.045), with no further increase thereafter. As eccentric load increased, the time course of moment development occurred earlier in the eccentric phase. This resulted in a 37% increase in eccentric knee extensor work from the 80% 1RM trial to the 120% 1RM trial (*P*<0.001). Neither hip nor ankle joints displayed further change in kinetics as eccentric load increased above 100% 1RM. Electromyographic activity during traditional squatting was ~15–30% lower in all eccentric trials than in concentric trials for all muscles. EMG plateaued between a load of 80–100% 1RM during the eccentric trials and did not increase with AEL. No significant differences in kinematics were found between Kineo and barbell squatting.

**Conclusions:**

The knee extensors appear to be preferentially loaded during AEL squatting. The greater work performed during the eccentric phase of the squat as eccentric load increased suggests greater total mechanical tension could be the cause of adaptations from AEL. Our data suggest that AEL should be programmed with a load of 120% of 1RM. Further studies are needed to confirm the longer-term training effects of AEL.

## Introduction

Increasing muscle force producing capacity is a primary goal for strength and conditioning (S&C) practice, as it can improve performance in a wide range of sporting activities [1, 2]. Enhanced force production can be achieved by increased neural drive and the addition of contractile material via skeletal muscle hypertrophy [3], both of which can be achieved with resistance training [4]. Skeletal muscle hypertrophy occurs as a result of an increased net-protein balance [5], which involves the activation of mTORC1 [6]. The stimulus from mechanical tension within the muscle has been highlighted as the primary mechanism by which hypertrophy occurs [7], with a dose-response relationship between the peak tension the muscle undergoes and the activation levels of mTORC1 [8]. These stimuli are detected by mechanosensors such as the kinase domain of the titin myofilament protein [7]. Furthermore, a total volume of mechanical tension, due to more work done by a muscle, has also been shown to increase markers of muscle protein synthesis [9]. However, highly trained athletes have a smaller physiological response (e.g., endocrine response) to resistance training, leading to an attenuation of skeletal muscle adaptation compared to untrained individuals [10]. Thus, highly resistance-trained individuals may require greater or novel stimuli to elicit adaptation. Therefore, S&C practitioners seek advanced training methods to facilitate continued adaptation, often by increasing the peak mechanical tension or volume of mechanical tension placed upon a muscle.

One such advanced training method is eccentric resistance training [11], whereby the duration or loading of the eccentric (muscle lengthening) phase of a given exercise is manipulated by applying loads above the individual’s concentric (muscle shortening) one-repetition maximum (1RM), or through the use of isoinertial or isovelocity devices such as the Kineo training system (kineo) (v7.0, GLOBUS, Italy) [12]. These training methods exploit the greater force producing capacity of eccentric compared to isometric or concentric muscle contractions [13]. During eccentric contractions, sarcomere length increases including active stretch of the spring-like titin myofilament increasing its stiffness [14], facilitating greater transference of forces through the sarcomere which can be detected by the kinase domain of titin, potentially leading to adaptation. The magnitude of eccentric force enhancement recorded is dependent on the conditions of measurement, with forces up to 80% greater in isolated muscle [15], and forces/moments up to 30% greater for single-joint movements [16] and 10% greater during multi-joint exercises [12]. These differences are likely due to an eccentric-specific neural activation strategy [17], and differences in neural activation during multi-joint movements [18]. To that end, the question arises whether sufficiently greater muscular forces are achieved during eccentric training to warrant the complexity of these training designs.

During traditional (TRAD) squatting (i.e., same absolute load for the concentric and eccentric phase), ground reaction forces have been reported to be greater during the concentric vs. the eccentric phase [19], given the load must be accelerated against gravity in the concentric phase. Consequently, the load during the eccentric phase in TRAD squatting is significantly below the maximum eccentric capacity, potentially under-loading the musculature and therefore providing sub-optimal mechanical tension to promote adaptation. However, the degree of this under-loading during the eccentric phase compared to the concentric phase is currently unknown.

One eccentric resistance training method that shows promise for overcoming the above limitations of TRAD squatting is accentuated-eccentric loading (AEL) [20] in which the load is greater during the eccentric phase than the concentric. By taking advantage of the direction-specific mechanical properties of muscle contraction, AEL can increase the peak and volume of mechanical tension experienced. Previous literature has highlighted promising results from AEL, with increases in both strength and hypertrophy [11], as well as maintained acute endocrine responses [21]. Thus, many elite S&C practitioners now adopt AEL into their training repertoire [22]. However, there is a dearth of information regarding how best to program AEL, especially considering a systematic review from 2017 found ~80% of eccentric research has been performed using single-joint methodologies [23], whereas multi-joint movements are typically used in applied practice (e.g., squatting). There is also a high variability between individuals in the absolute and relative magnitude of maximum eccentric forces produced during squatting [12]. Collectively, these issues make it difficult to produce scientifically justified AEL training recommendations.

Harden *et al*. [24] explored the issue of eccentric under-loading using a pneumatic leg-press to deliver loads equivalent to 110, 130, & 150% of isometric force, and found that a greater eccentric load results in an increase in eccentric ground reaction force [24]. Likewise, Sarto *et al*. [25] demonstrated that an AEL load of 150% during a leg-press results in a 31% increase in quadriceps muscle activity compared to a TRAD eccentric load of 80% of 1RM. Taken together, these studies indicate a greater loading of the lower limb musculature during AEL. However, it is uncertain how this would translate to squatting, due to the differences in kinematics and muscle activity between the squat and leg-press [26]. One of the few studies that has examined the eccentric phase of the squat with AEL [27] found that this resulted in a greater eccentric work. Unfortunately, this was only assessed with one AEL load (105% of concentric 1RM) and did not investigate individual joint kinetics [27], and therefore comprehensive training recommendations cannot be established.

In order to produce comprehensive training recommendations for AEL squatting, it is necessary not only to understand the total load that can be lifted, but also to understand the joint contributions, as not all joints are loaded equally during multi-joint movements. Additionally, the loading experienced in each phase of the squat may vary due to changes in squatting technique. During the eccentric phase athletes may alter their strategy to control the load and may not produce maximum effort throughout the full range of descent [28], and thus descent velocity can vary [29]. These changes may affect the rate of moment development, work, and peak joint moment exerted by the lower limb muscles, with work being an indicator of the total volume of mechanical tension, and peak joint moment an indicator of peak mechanical tension experienced by a muscle. Therefore, in order to identify optimal AEL protocols, a range of AEL parameters needs to be assessed. Application of squatting loads greater then 1RM can be risky and challenging, or requires specialist equipment such as the Kineo, which we have previously demonstrated to be safe and effective for this purpose [12]. However, we also need to understand whether the squat technique when using the Kineo differs to those of typical squat variations (e.g., barbell back squat/front squat.

Therefore, the primary objectives of this study were to study the application of AEL during squatting and to: 1) determine how the eccentric joint moments and work of the lower limb joints change with the magnitude of eccentric load; 2) determine how the concentric and eccentric joint moments and work of the lower limbs differed during TRAD loading; 3) establish whether/how lower limb peak joint moments and work from the AEL trials differ from those achieved during commonly prescribed TRAD loads used for resistance training. Secondary objectives were to investigate whether any changes in joint moments and work are accompanied by changes in muscle activity or changes in joint kinematics, and if the squat kinematics differ between barbell variations and the Kineo.

It was hypothesised that: 1) as eccentric load increased, the joint moment and work of the lower limbs would increase; 2) concentric joint moments and work of the lower limbs would be greater than eccentric joint moments and work during TRAD loading; 3) eccentric joint moments and work during AEL would exceed those during the concentric phase of TRAD; and 4) an increased joint moment would be accompanied by an increase in EMG activity.

## Materials and methods

### Participants

Nine male participants were recruited for this study (age; 24 ± 2 years, body mass; 81.2 ± 8.6 kg, height; 178 ± 5 cm). This sample size exceeded the minimum participant sample size (n = 7) determined using joint moment data from previous research [30] with power and alpha levels set to 0.8 and 0.05, respectively. All participants had completed at least twelve months of resistance training prior to this study and had a mean relative barbell back squat 1RM of 1.71 ± 0.17 body mass. Prior to commencement, participants were informed of the study procedures and gave written informed consent. The study was approved by the Liverpool John Moores University research ethics committee (19/SPS/038).

### Experimental protocol

All squatting trials (both TRAD and AEL) were performed on the Kineo Training System (Kineo) (V7, GLOBUS, Italy), which is a motorised cable pulley system that facilitates AEL squatting via automatic load adjustments at pre-defined ranges of motion. We have previously shown the Kineo to be reliable, accurate and safe in application of accentuated-eccentric loads during squatting [12]. Participants reported to the Liverpool John Moores laboratories on three occasions. The first visit was used for familiarisation to the Kineo and AEL squatting. During the second visit, each participant’s concentric 1RM squat on the Kineo was measured. Experimental data were collected on the third visit, consisting of kinetics, 3D kinematics and electromyography (EMG) during TRAD and AEL Kineo squatting. Each session began with a standardised warmup following the RAMP protocol [31].

### Familiarisation and squat set up

**Fig 1:**
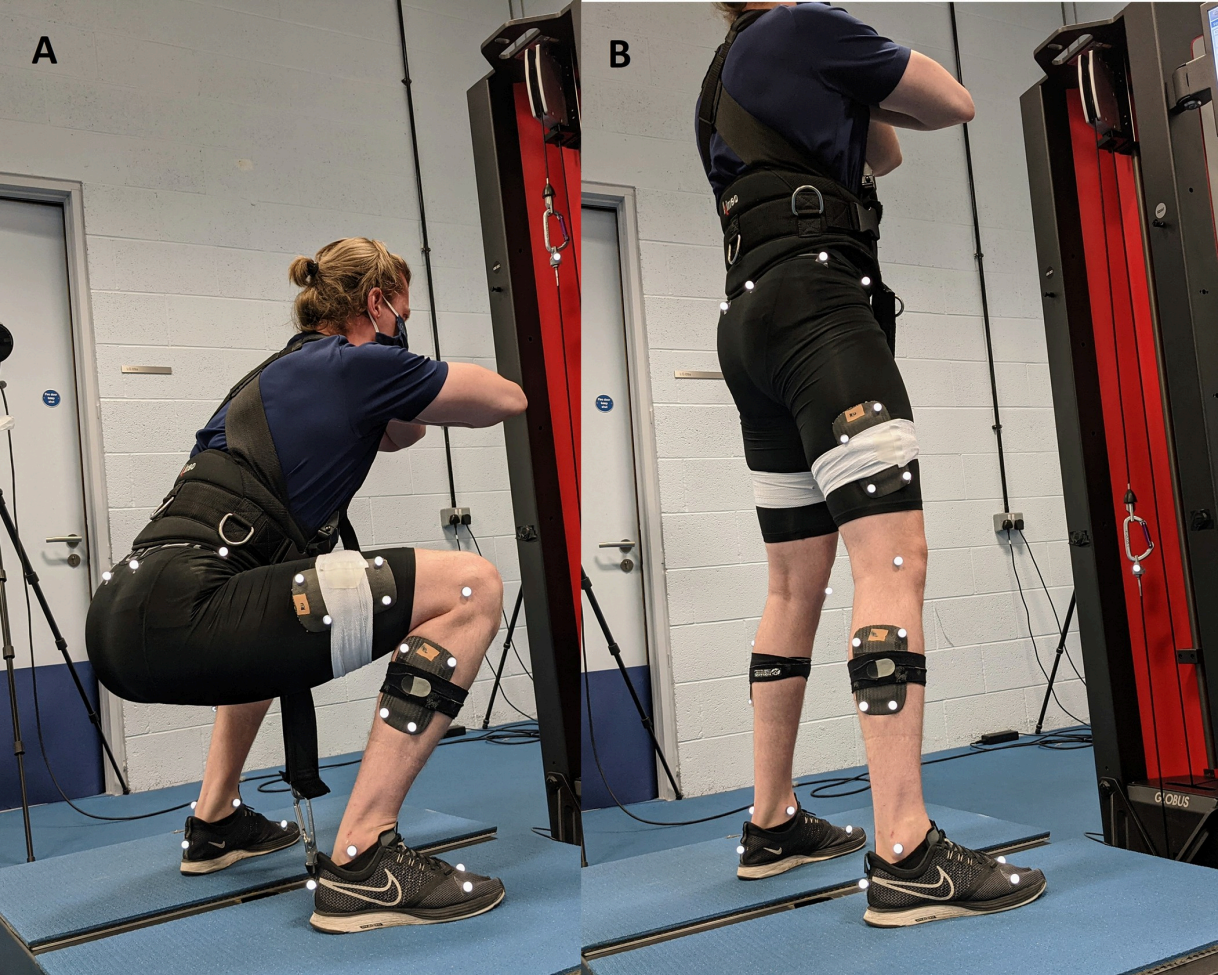
Example of participant performing a squat utilising the Kineo Training System. The participant is attached to the machine utilising a shoulder/hip harness, which is then attached to a cable protruding from the floor beneath the participant. **A)** Start of eccentric phase, **B)** end of eccentric phase/start of concentric phase.

Participants were fitted with a shoulder/hip harness, adjusted for goodness of fit, before being attached to the Kineo via a cable (**Fig 1)**. Range of motion was determined, so that the eccentric phase commenced until the participant had squatted down to a depth at which the top of the thighs were parallel to the ground. An audible signal was given when this depth was attained and confirmed via 3D motion analysis. The concentric phase began immediately after the end/completion of the eccentric phase and until the participant had fully extended the hips and knees. The corresponding cable positions were programmed and saved within the Kineo software control system to facilitate automatic load changes for AEL squatting during the experimental testing. Participants finished the familiarisation session with several sets of TRAD and AEL squatting ranging from 20–150% of estimated concentric 1RM, to become familiar with the automatic load adjustments during AEL.

### One-repetition maximum testing

Participants reported to the laboratories in a fed and hydrated state. Body mass (± 0.1 kg) and height (± 0.5 cm) were measured (SECA 704/202, Germany). Participants then performed a standardised warmup, finishing with several progressively heavier squats on the Kineo, following protocols of the National Strength and Conditioning Association [32]. Participants were then allowed a maximum of five attempts to establish a TRAD squatting 1RM using isotonic loads applied from the Kineo, adhering to the technique outlined in the familiarisation, with 3–5 minutes passive rest between attempts.

### Kinetic, kinematic, and electromyography testing

On the third visit (5–7 days post 1RM), participants reported to the laboratory at a similar time of day to that at which they performed the 1RM testing. Upon completion of the standardised warmup, participants were fitted with reflective markers and surface electromyography electrodes (BlueSensor, Ambu, Denmark).

Thirty-six spherical reflective markers were used to define and track the lower limb segments. This included a modified CODA pelvis marker set to define the pelvis segment with additional tracking markers on the iliac crest to aid in pelvis tracking and to overcome ASIS marker occlusion during deep hip flexion. The remaining markers were placed on the lateral & medial femoral epicondyles, lateral & medial malleoli, heel, and 1^st^ & 5^th^ metatarsals to define the thigh, shank, and foot segments. Additionally, rigid four-marker cluster sets were placed on the lateral thighs and shanks to aid in thigh and shank tracking. Joint centres of the hip and knee were identified by functional movement trials that isolated movements of those joints, and were calculated using the Gillette algorithm [33]. Surface electromyography electrodes were placed on the gluteus maximus, vastus lateralis, biceps femoris, and gastrocnemius medialis according to the SENIAM guidelines [34]. Prior to electrode placement, the skin was prepared by shaving and abrading to enhance signal quality.

Participants performed ten trials of squatting (five TRAD, five AEL) in a randomised order. TRAD squatting applied the same absolute load for both the concentric phase and eccentric phase (20%, 40%, 60%, 80%, and 100% 1RM). AEL squatting applied an increased load (compared to concentric 1RM) in the eccentric phase (110%, 120%, 130%, 140%, and 150% 1RM) whilst the concentric load remained at 60% 1RM for all AEL trials. A load of 60% 1RM was chosen for the concentric trials based upon pilot testing, as it enabled enough preload to enable maximal eccentric contractions [16], whilst minimising excess fatigue. Load adjustment between the eccentric and concentric phase for AEL trials was performed automatically by the Kineo once the programmed transition point had been reached. All trials were performed with three repetitions, interspersed by five minutes passive recovery. The average of the three trials was used for data analyses.

### Data acquisition and analyses

During all trials, ground reaction forces were collected from two force plates sampling at 1500 Hz (9287c, Kistler, Switzerland), amplified (9865, Kistler, Switzerland) and converted to a digital signal. Reflective markers were tracked at 200 Hz using six 3D motion capture cameras (Opus 3 series, Qualisys, Sweden). Electromyographic signals were sampled at 1500 Hz and transmitted wirelessly (Research DTS, Noraxon, USA). All force, motion and EMG data were recorded synchronously in Qualisys Track Manager (Qualisys, Sweden), before being exported to Visual 3D (C-Motion, USA) for analyses. Force and motion data were processed with a lowpass 4^th^ order Butterworth filter, with a cut off frequency of 6 Hz. EMG data was band pass filtered between 10 and 250 Hz and root mean squared with a moving average of 100 ms.

Joint range of motion (°) and joint velocity (°·s^-1^) during the concentric and the eccentric phases were quantified. Inverse dynamics calculations were used to calculate joint moments of the hip, knee and ankle during the concentric and eccentric phases of the squat, which were normalised to body mass (N·m·kg^-1^). Integration of the joint power curve allowed for the calculation of eccentric and concentric joint work (J). Joint work was reported as an absolute magnitude, irrespective of direction (+ or -) for ease of comparison and graphical representation. Electromyography data were analysed for peak EMG, and total integrated EMG of the eccentric and concentric phases of each joints individual range of motion. All EMG data were normalised to the equivalent measure obtained during the concentric TRAD 100% trial.

### Statistical analyses

Mauchly’s test for sphericity, Levene’s test for homogeneity of variance, and Shapiro-Wilk’s test for normality were performed on all data. Greenhouse-Geisser corrections were used on data that violated the assumption of sphericity. All data were normally distributed (*P* = 0.145–0.814) and had a homogeneity of variance (*P* = 0.157–0.987). To assess study objective one, a one-way repeated measures ANOVA was used to determine if eccentric load (20 to 150% 1RM) had an effect on the kinetics, kinematics, and muscle activity during the eccentric phase of the squat. To assess study objective two, a two-way repeated measures ANOVA was used to compare the kinetics, kinematics, and muscle activity during the concentric and eccentric phase of the squat during TRAD loading (20 to 100% 1RM). Study objective three was subsequently assessed with a one-way repeated measures ANOVA to compare the joint moment and work from the AEL trials that lead to the greatest eccentric kinetics to the concentric 80% and 100% trials. Bonferroni post-hoc analyses were used in all tests where appropriate. Effect sizes were calculated for all ANOVA tests using ω^2^, with values of 0.01, 0.06, and 0.14 indicating a small, medium and large effect size, respectively [35]. Additionally, a paired-samples t-test was used to perform a comparison between the 80% 1RM and 120% 1RM trial. A Cohen’s *d* effect size was calculated with values of 0.2, 0.5, and 0.8 representing a small, medium, and large effect size, respectively.

Coefficient of variation (CV) was used to identify repetition-to-repetition reliability. Peak joint moment (CV = 2.6–4.1%), joint work (CV = 1.8–2.4%), joint velocity (CV = 4.7–5.2%) and EMG (CV = 7.2–18.6%) had acceptable reliability and was similar to previous literature [30]. For all data, statistical significance was assessed with an alpha level of 0.05. All analyses were performed in Statistical Package for the Social Sciences (SPSS version 27, IBM, USA).

### Comparison between Kineo and barbell squatting

A secondary cohort of resistance trained males (age: 25 ± 2 years, weight: 78 ± 7 kg, height: 179 ± 6 cm; UREC code: 21/SPS/035) performed barbell back squat, barbell front squat, and Kineo squat under 50, 85, and 100% of body mass, in a randomised order. Movement kinematics was assessed in the same way as described earlier and EMG was collected from gluteus maximus and vastus lateralis. For a detailed description of the methodology please see the S1 File.

## Results

**Fig 2:**
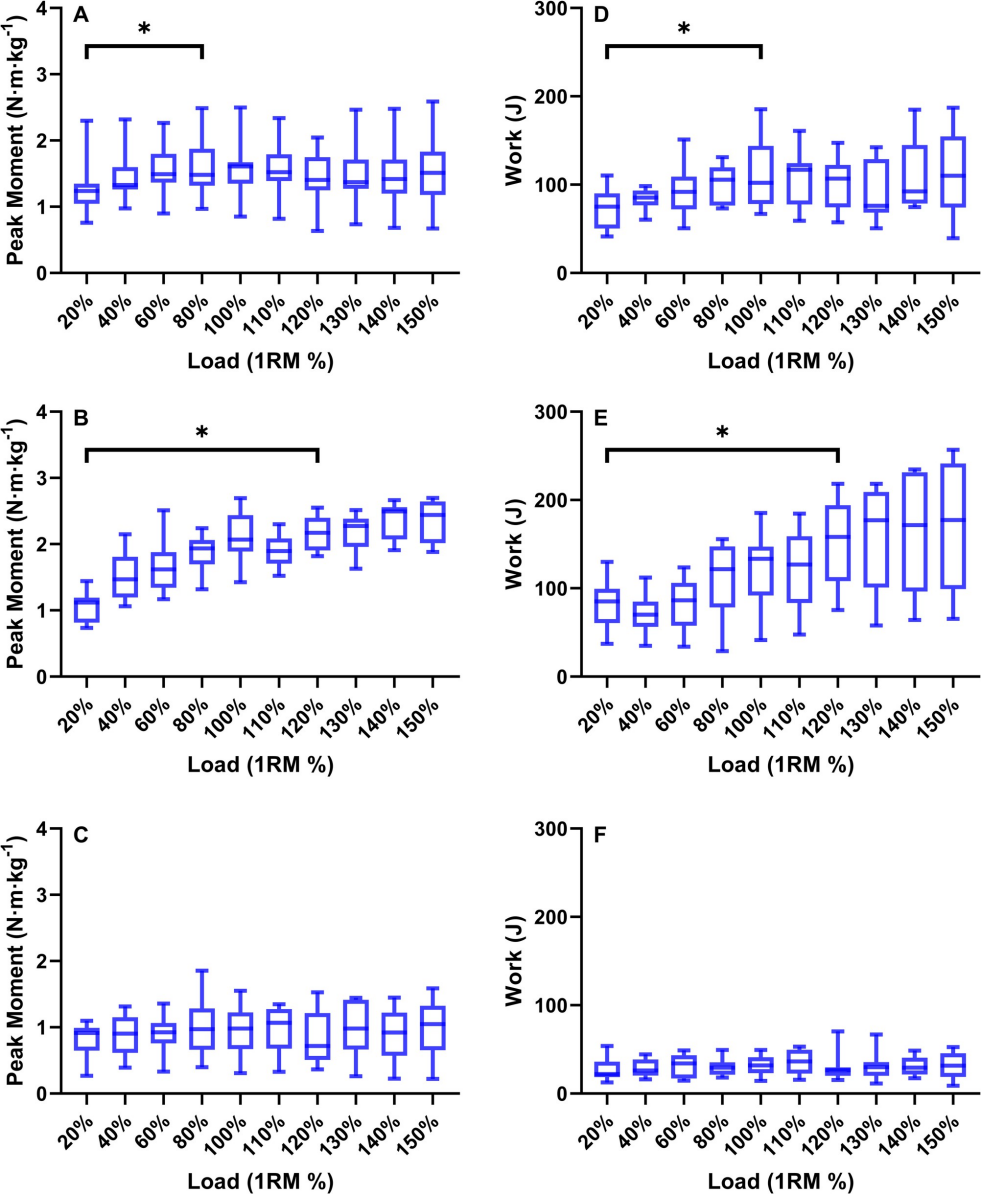
Box-plots (median ± IQR) displaying the eccentric peak moment (N·m·kg^-1^) and work (J) for the hip (**A** and **D**), knee (**B** and **E**), and ankle extensors (**C** and **F**) during the eccentric phase of the squat with an external load of 20–150% 1RM. * = significant increase.

There was a significant effect of loading condition on the peak joint moments in the eccentric phase for the hip (F = 2.773, *P* = 0.007, ω^2^ = 0.17) (**Fig 2A**) and knee (F = 16.408, *P*<0.001, ω^2^ = 0.61) (**Fig 2B**), but not on the ankle (F = 0.254, *P* = 0.985, ω^2^ = -0.08) (**Fig 2C**). Post-hoc testing revealed that there was a plateau in peak eccentric moment at a load of 80% for the hip (*P* = 0.039), and at 120% for the knee (*P* = 0.045).

Analyses of eccentric joint work found that there was a significant effect of loading only on the knee (F = 5.438, *P*<0.001, ω^2^ = 0.31) (**Fig 2E**), with no effect on the hip (F = 1.2, *P* = 0.307, ω^2^ = 0.02) (**Fig 2D**) or ankle (F = 0.171, *P* = 0.996, ω^2^ = -0.09) (**Fig 2F**). Post-hoc testing of the eccentric knee work revealed a plateau at a load 120% (P = 0.022).

**Fig 3:**
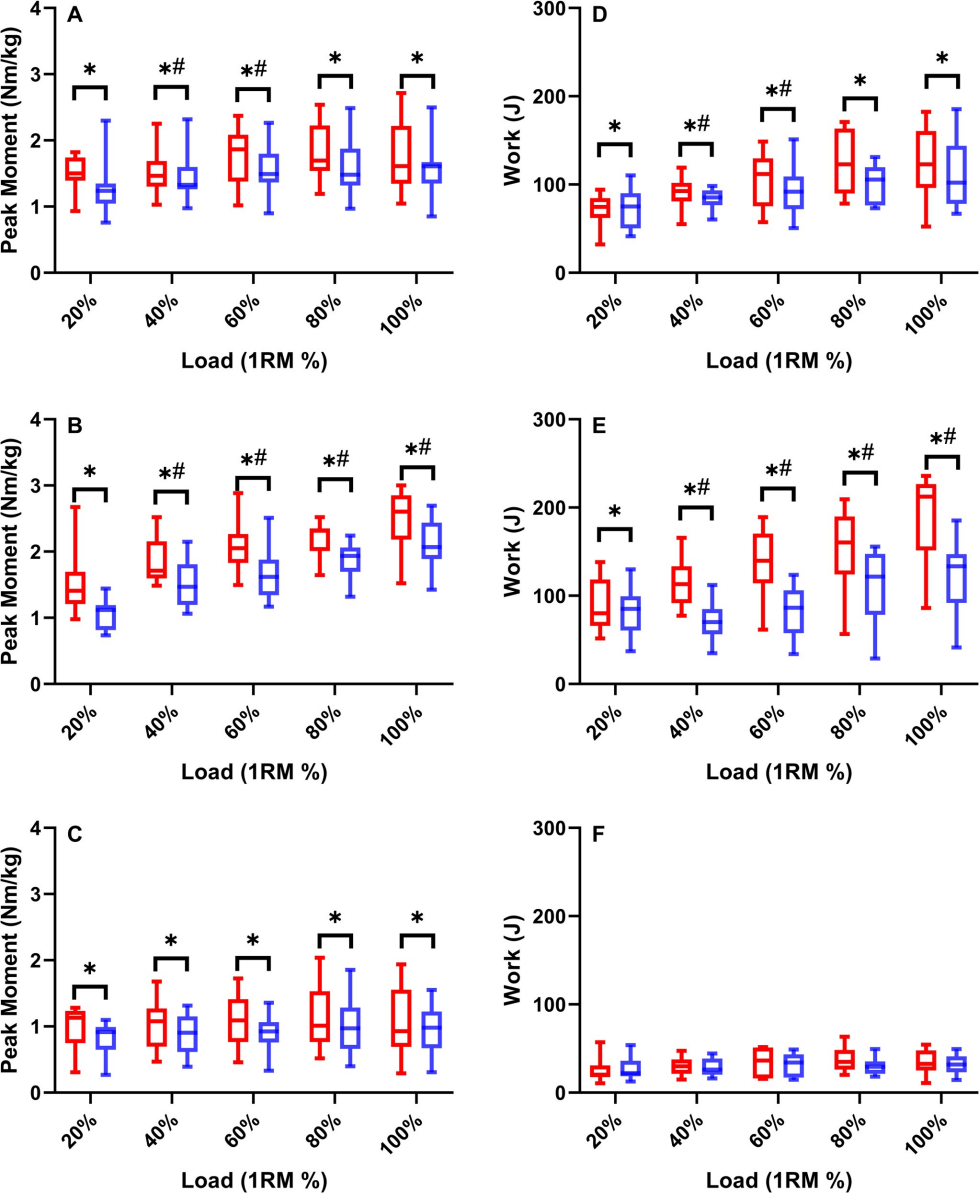
Box-plots (median ± IQR) displaying the concentric (red bars) and eccentric (blue bars) peak moment (N·m·kg^-1^) and work (J) for the hip (**A** and **D**), knee (**B** and **E**), and ankle extensors (**C** and **F**) during TRAD (20–100%) squatting. * = Eccentric joint kinetics (moment or work) is statistically smaller than concentric joint kinetics at the same given load. # = Joint Kinetics is statistically different to the preceding trial.

During TRAD squatting, the peak joint moments were greater in the concentric than eccentric phase for the hip (F = 3.982, *P* = 0.049, ω^2^ = 0.03), knee (F = 24.729, *P*<0.001, ω^2^ = 0.13) and ankle (F = 3.691, *P* = 0.044, ω^2^ = 0.03) (**Fig 3A–3C**). Similarly, joint work was greater in the concentric than eccentric phase for the hip (F = 3.783, *P* = 0.045, ω^2^ = 0.02) and knee (F = 31.58, *P*<0.001, ω^2^ = 0.19). However, no difference was found between the concentric and eccentric ankle work (F = 0.819, *P* = 0.368) (**Fig 3D–3F**). Post-hoc analyses identified that the concentric and eccentric knee extension peak moment and work increased with load up to 100% 1RM (*P* = 0.003). Concentric and eccentric hip extension moment and work did not significantly increase past 60% (*P* = 0.039), and the ankle extensors saw no effect of loading on either peak moment or work for either the concentric or eccentric phase (*P* = 0.084).

**Fig 4:**
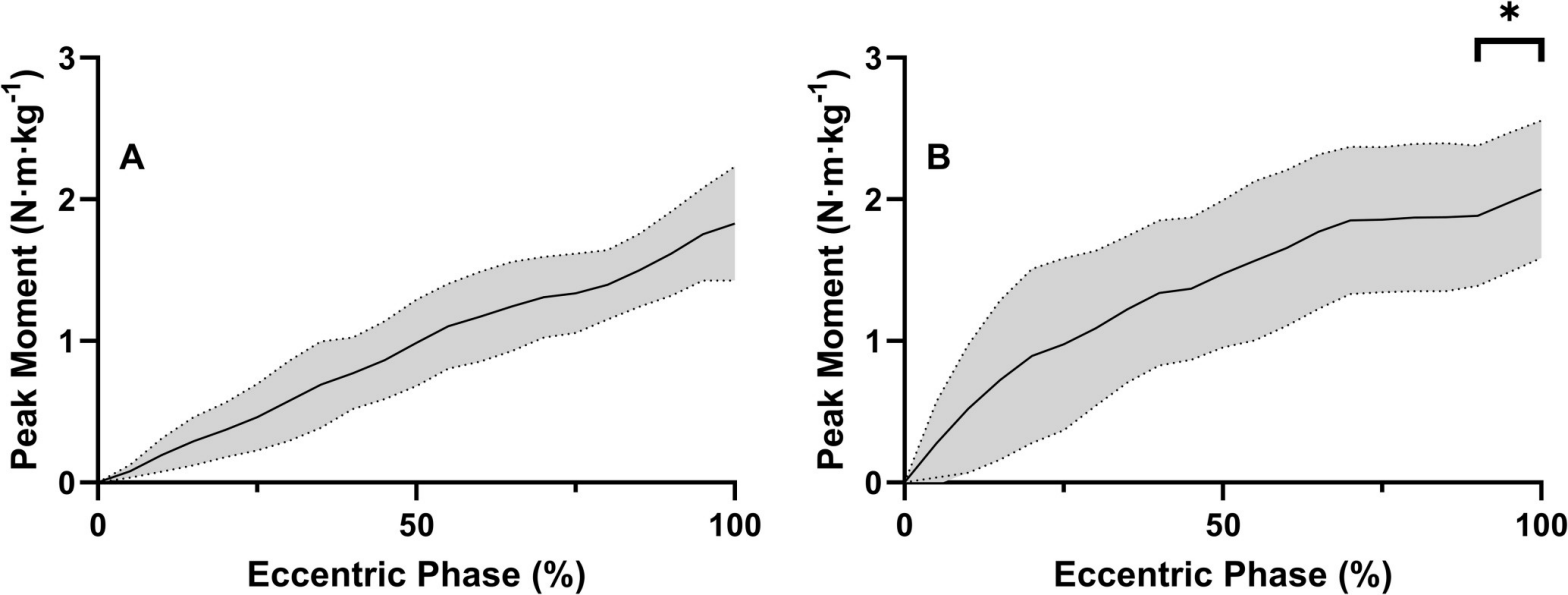
Mean ± SD eccentric knee extension moment (N·m·kg^-1^) over the eccentric phase duration (%) graph, demonstrating the increase in early rate of moment development as eccentric load increased from 80% 1RM (**A**), to 120% 1RM (**B**). * = significant increase in peak moment.

As both eccentric knee moment and work plateaued at 120%, these data were compared to the concentric knee moments and work at 80% and 100% (moment: F = 2.775, *P* = 0.05, ω^2^ = 0.1, work: F = 2.251, *P* = 0.125, ω^2^ = 0.08). It was found that peak knee moment in the eccentric phase of the AEL 120% trial was significantly less than in the concentric phase of 100% (*P* = 0.042), but not significantly different from concentric 80% trial (*P* = 0.839). Eccentric knee work at 120% was significantly greater than at 80% (*t* = -6.444, *P*<0.001, Cohen’s *d* effect size = 0.81) (**Fig 4**).

The knee moment-time graphs during the eccentric phase (normalised to eccentric phase duration) (**Fig 4**) reveal changes in the time course of moment development as load increased, which helps to explain the effects of load on peak moment and work during the AEL trials. As external load increased from 80% to 120% 1RM, eccentric knee extensor peak moment increased (17%) and then plateaued, with a distinct peak occurring towards the end of the range of motion. Furthermore, with each increase in load, moment development in the first half of the movement was greater, resulting in a 37% increase in work from the 80% 1RM trial to the 120% 1RM trial (**Fig 4**).

**Fig 5:**
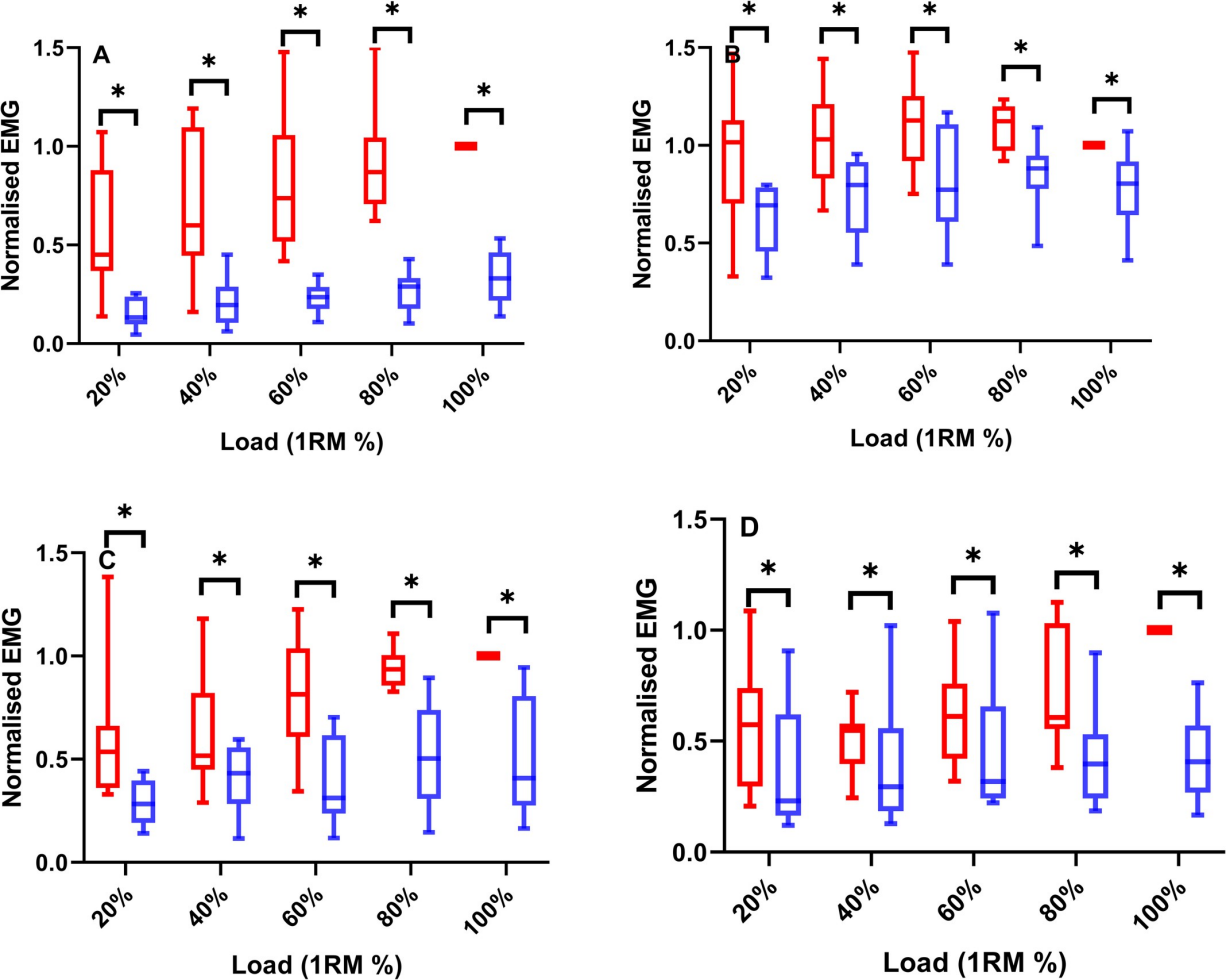
Box-plots (median ± IQR) displaying the concentric (red bars) and eccentric (blue bars) normalised EMG for the gluteus maximus (**A**), vastus lateralis (**B**), biceps femoris (**C**) and gastrocnemius medialis (**D**) during TRAD (20–100%) squatting. * = Eccentric muscle activity is significantly smaller than concentric muscle activity at the same given load.

**Fig 6:**
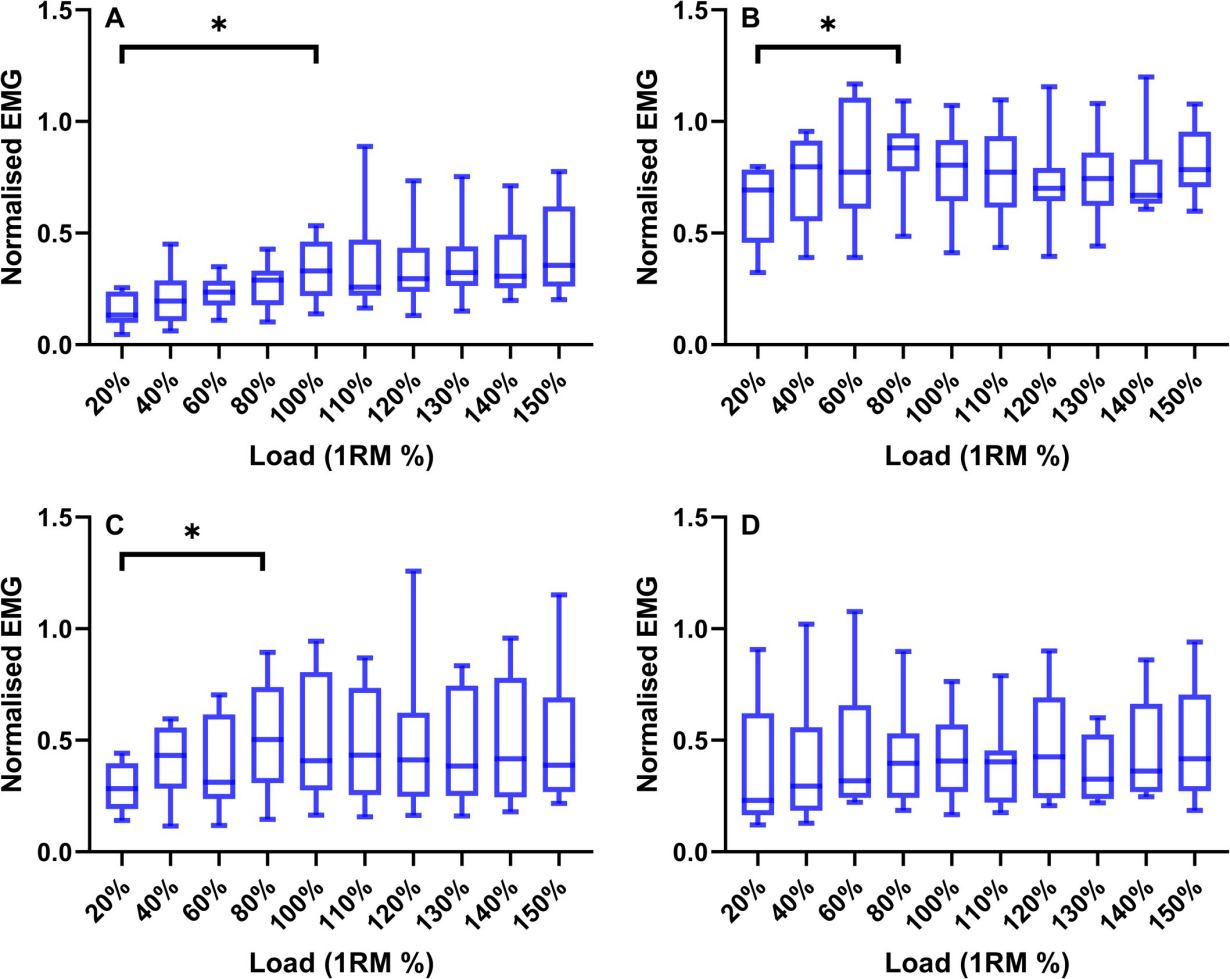
Box-plots (median ± IQR) displaying the eccentric normalised EMG for the gluteus maximus (**A**), vastus lateralis (**B**), biceps femoris (**C**) and gastrocnemius medialis (**D**) during the eccentric phase of the squat with an external load of 20–150% 1RM. * = significant increase.

Analyses of EMG activity identified greater peak magnitudes during concentric than eccentric phases during TRAD squatting across all loads; gluteus maximus (F = 51.952, *P*<0.001, ω^2^ = 0.58), vastus lateralis (F = 29.81, *P*<0.001, ω^2^ = 0.27), biceps femoris (F = 20.852, *P* = 0.002, ω^2^ = 0.35), and gastrocnemius medialis (F = 18.545, *P*<0.001, ω^2^ = 0.14) (**Fig 5**). Additionally, eccentric loading had an effect on EMG activity, with an increase in activity as load increased up to 100% for the gluteus maximus (F = 4.069, *P*<0.001, ω^2^ = 0.23), 80% for the vastus lateralis (F = 2.165, *P* = 0.033, ω^2^ = 0.10) and biceps femoris (F = 2.754, *P* = 0.007, ω^2^ = 0.14), whilst there was no effect of load on the gastrocnemius medialis activity (F = 1.00, *P* = 0.447) (**Fig 6**).

The joint angular ranges of motion kinematics did not differ between loads for the hip (F = 0.274, *P* = 0.98), knee (F = 0.276, *P* = 0.979), or ankle joints (F = 0.155, *P* = 0.998) (**Table 1**). The joint angle at which the peak moment occurred, during both the concentric and eccentric phases, was not different between loads for the hip (F = 7.03, *P* = 0.426), knee joints (F = 5.228, *P* = 0.052), or ankle joint (F = 0.610, *P* = 0.658). However, the peak concentric ankle joint moment occurred in a significantly more dorsi-flexed position than the peak eccentric moment (34 ± 1° vs 26 ± 1°) (*P* = 0.006) (**Table 1**). Lastly, concentric joint angular velocity was greater for all joints, at all loads, than eccentric joint angular velocity (**Table 2**). For the hip (F = 17.219, *P*<0.001), velocity during concentric and eccentric phases reduced as load increased up to 80% 1RM, after which it plateaued. Similar results were found for the ankle (F = 8.516, *P*<0.001) with a plateau after 60%. However, the knee joint angular velocity only showed a plateau after 100% 1RM (F = 22.837, *P*<0.001). There was no further decrease in angular velocity as eccentric load increased above 100% (*P* = 0.698 to 0.99).

**Table 1:** Joint angle kinematics (mean ± SD) for the hip, knee, and ankle joints during the concentric and eccentric phases of the squat with external loads ranging from 20% to 150% of concentric one-repetition maximum. * = Joint angle during the eccentric phase is statistically smaller than during the concentric phase at the same given load.

			Loading Condition (percentage of concentric one repetition maximum) (%)									
			20%	40%	60%	80%	100%	110%	120%	1305	140%	150%
Joint Angle at Peak Moment (°)	Hip	Concentric	84 ± 17°	83 ± 18°	76 ± 16°	79 ± 14°	81 ± 13°					
		Eccentric	80 ± 11°	86 ± 14°	86 ± 11°	86 ± 11°	77 ± 10°	77 ± 13°	74 ± 12°	77 ± 14°	77 ± 10°	80 ± 14°
	Knee	Concentric	102 ± 11°	104 ± 10°	101 ± 7°	101 ± 8°	99 ± 14°					
		Eccentric	104 ± 10°	107 ± 9°	105 ± 6°	105 ± 10°	103 ± 10°	101 ± 8°	102 ± 9°	98 ± 7°	101 ± 9°	103 ± 9°
	Ankle	Concentric	37 ± 3°	36 ± 4°	34 ± 4°	32 ± 5°	30 ± 4°					
		Eccentric	*23 ± 7°	*26 ± 12°	*24 ± 8°	29 ± 9°	*27 ± 8°	25 ± 7°	23 ± 7°	23 ± 7°	26 ± 9°	23 ± 5°
Range of Motion (°)	Hip		89 ± 12°	93 ± 14°	91 ± 9°	93 ± 11°	89 ± 10°	88 ± 10°	89 ± 10°	88 ± 10°	90 ± 10°	90 ± 10°
	Knee		112 ± 8°	112 ± 8°	111 ± 8°	111 ± 9°	108 ± 6°	108 ± 8°	109 ± 8°	108 ± 7°	109 ± 8°	110 ± 8°
	Ankle		41 ± 3°	40 ± 3°	40 ± 2°	40 ± 3°	40 ± 3°	40 ± 3°	40 ± 3°	40 ± 2°	40 ± 2°	40 ± 3°

**Table 2:** Joint angular velocity kinematics (mean ± SD) for the hip, knee, and ankle joints during the concentric and eccentric phases of the squat with external loads ranging from 20% to 150% of concentric one-repetition maximum. * = eccentric angular velocity is statistically slower than concentric angular velocity at the same given load. # = Angular velocity is statistically different to the preceding trial.

			Loading Condition (percentage of concentric one-repetition maximum) (%)									
			20%	40%	60%	80%	100%	110%	120%	1305	140%	150%
Peak Angular Velocity (°·s^-1^)	Hip	Concentric	200 ± 83°·s^-1^	#180 ± 65°·s^-1^	#194 ± 66°·s^-1^	#182 ± 57°·s^-1^	185 ± 55°·s^-1^					
		Eccentric	*91 ± 30°·s^-1^	*#113 ± 41°·s^-1^	*#81 ± 17°·s^-1^	*#102 ± 29°·s^-1^	*84 ± 27°·s^-1^	77 ± 35°·s^-1^	80 ± 22°·s^-1^	80 ± 30°·s^-1^	79 ± 23°·s^-1^	74 ± 22°·s^-1^
	Knee	Concentric	257 ± 74°·s^-1^	#251 ± 62°·s^-1^	#260 ± 62°·s^-1^	#250 ± 55°·s^-1^	256 ± 42°·s^-1^					
		Eccentric	*126 ± 26°·s^-1^	*#142 ± 43°·s^-1^	*#116 ± 32°·s^-1^	*115 ± 32°·s^-1^	*#101 ± 31°·s^-1^	#91 ± 25°·s^-1^	98 ± 25°·s^-1^	95 ± 28°·s^-1^	104 ± 29°·s^-1^	97 ± 28°·s^-1^
	Ankle	Concentric	107 ± 41°·s^-1^	#102 ± 23°·s^-1^	103 ± 25°·s^-1^	101 ± 28°·s^-1^	105 ± 26°·s^-1^					
		Eccentric	*48 ± 16°·s^-1^	*#57 ± 16°·s^-1^	*#49 ± 13°·s^-1^	*46 ± 12°·s^-1^	*40 ± 11°·s^-1^	36 ± 8°·s^-1^	38 ± 11°·s^-1^	38 ± 11°·s^-1^	43 ± 12°·s^-1^	39 ± 9°·s^-1^
Average Angular Velocity (°·s^-1^)	Hip	Concentric	94 ± 29°·s^-1^	#86 ± 24°·s^-1^	#77 ± 18°·s^-1^	#68 ± 17°·s^-1^	#60 ± 12°·s^-1^					
		Eccentric	*52 ± 15°·s^-1^	*#57 ± 20°·s^-1^	*#48 ± 15°·s^-1^	*#44 ± 13°·s^-1^	*41 ± 13°·s^-1^	39 ± 40°·s^-1^	42 ± 15°·s^-1^	38 ± 14°·s^-1^	40 ± 17°·s^-1^	41 ± 16°·s^-1^
	Knee	Concentric	128 ± 28°·s^-1^	#117 ± 21°·s^-1^	#107 ± 16°·s^-1^	#92 ± 14°·s^-1^	#79 ± 14°·s^-1^					
		Eccentric	*74 ± 18°·s^-1^	*#79 ± 21°·s^-1^	*#69 ± 16°·s^-1^	*#63 ± 16°·s^-1^	*#57 ± 16°·s^-1^	55 ± 18°·s^-1^	60 ± 20°·s^-1^	54 ± 20°·s^-1^	56 ± 24°·s^-1^	53 ± 23°·s^-1^
	Ankle	Concentric	36 ± 10°·s^-1^	#32 ± 5°·s^-1^	#30 ± 5°·s^-1^	#26 ± 4°·s^-1^	24 ± 5°·s^-1^					
		Eccentric	*19 ± 4°·s^-1^	*21 ± 4°·s^-1^	*19 ± 3°·s^-1^	*18 ± 3°·s^-1^	*17 ± 5°·s^-1^	17 ± 4°·s^-1^	18 ± 5°·s^-1^	16 ± 5°·s^-1^	16 ± 6°·s^-1^	16 ± 5°·s^-1^

### Comparison between Kineo and barbell squatting

**Fig 7:**
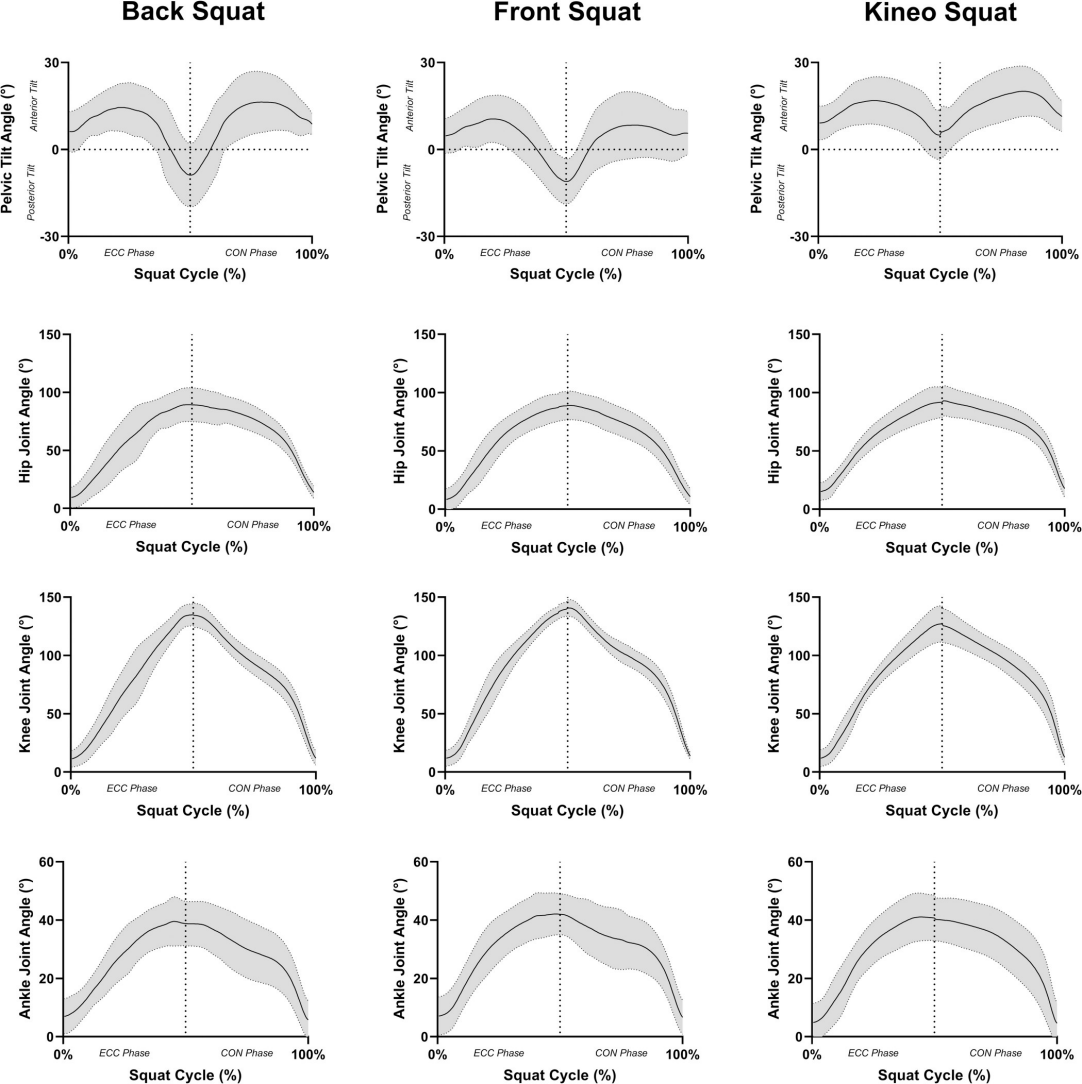
Mean ± SD joint angle (°) during the eccentric phase and concentric phase of the barbell back squat, barbell front squat, and Kineo squat with an external load of 100% of bodyweight. Positive pelvic tilt angle is representative of anterior pelvic tilt, with a negative angle being representative of posterior pelvic tilt. Positive ankle angle is representative of dorsiflexion, with a negative angle being representative of plantar flexion.

Analyses of joint ranges of motion found that there was no effect of squatting variation on the range of motion for the hip (F = 0.338, *P* = 0.719), knee (F = 3.365, *P* = 0.109), or ankle joints (F = 1.295, *P* = 0.281). However, there was a medium effect of squatting variation on pelvis range of motion (F = 4.127, *P* = 0.039, ω^2^ = 0.08), with the Kineo squat (11±8°) having a significantly smaller pelvic range of motion than both the barbell back squat (21±6°) and barbell front squat (20±5°) (**Fig 7)**. External load did not have any effect on joint range of motion (*P* = 0.09–0.754). For analyses of joint velocities and muscle activity please see the S1 File.

## Discussion

In this study we have established the joint kinetics, mechanical loading and kinematic characteristics of traditional and AEL squatting. Supporting our first hypothesis, we discovered that peak eccentric knee extensor moments occurred at 120% of 1RM, plateauing with further increases in external squat load. The hip and ankle extensors showed no increase above 100% 1RM in eccentric joint moment or work during AEL squatting. Although only small increases in peak knee joint moment were observed, eccentric moment development occurred earlier during the eccentric phase as eccentric load increased (**Fig 4**), contributing to a continuous increase in knee joint work up to 150% of 1RM. Furthermore, the vastus lateralis appears to experience the greatest eccentric EMG activity (**Fig 5/6**) (relative to concentric 1RM activity). Taken together, these results indicate that the knee extensors, rather than the hip extensors, contribute most to the increasing squat load and experience the greatest loading [stimulus] in the form of peak joint moment and joint work during AEL squatting.

A secondary component of this study was to investigate whether the kinematics and muscle activity during squats performed on the Kineo differed from barbell squatting. Our findings suggest that the Kineo squat involves similar ranges of motion to both the barbell back squat and the barbell front squat for the hip, knee, and ankle joints. However, the Kineo squat presented a reduced pelvic tilt range of motion, that resulted in less posterior pelvic tilt when the thighs were parallel to the ground. Muscle activity during the Kineo squat was more akin to the front squat than the back squat, but the difference in muscle activity was small (difference in normalised EMG = 8%). Therefore, the findings obtained from the primary focus of this study should be transferable to the barbell squatting variants, providing loading is applied in a safe manner.

The first aim of this study was to identify whether increased squat loading would result in an increased eccentric joint moment and work. We can accept the first hypothesis, since there was an increase in both knee extensor moment and work as eccentric load increased with a plateau in knee extensor moment occurring at 120% 1RM, and a plateau in hip extensor moment occurring at 80% 1RM. This contrasts with what is known about the concentric phase of squatting, in which the hip extensor moment increases to a greater extent than the knee extensors as load is increased [30, 36]. Although this may be explained by differences in kinematics and muscle activation, we found no changes in joint ranges of motion or velocities as eccentric load increased above 100% (i.e., AEL). However, during heavy concentric squatting (70% 1RM) the forwards inclination of the trunk can increase by ~16° compared to lighter loads (30% 1RM) [37], increasing the moment arm of the centre of mass about the hips and reducing the moment arm at the knee, explaining joint-specific contributions in those studies [30, 36]. Therefore, in the present study it could be that the preferential loading of the knee extensors during AEL is a result of the participants having not altered their kinematics and joint dynamics with increasing eccentric load by keeping the trunk more vertical. The preferential loading at the knee during AEL squatting is further supported by the EMG data (**Fig 6**) which is consistent with previous literature [38] showing EMG activity of the hip extensors is lower than the activity of the knee extensors during eccentric squatting. However, caution must be taken when extrapolating the result of this study to barbell squatting. We have shown that the barbell and Kineo squat have similar kinematics and muscle activity in the lower limbs under traditional loading conditions. For this data to be transferable to barbell squatting, the participants must be able to maintain their kinematics during the eccentric phase during AEL, which may be a more complex movement pattern due to the high centre of mass with the barbell being positioned on the posterior deltoids. Therefore, AEL squatting may only be applicable to well-trained individuals.

Supporting our second hypothesis, the joint moments in the concentric phase were 11–20% greater than in the eccentric phase (**Fig 3**). This difference was similar for the hip, knee and ankle extensors, therefore providing more evidence that during TRAD squatting the eccentric phase is underloaded and may therefore be sub-optimal as a training stimulus, considering peak eccentric ground reactions forces during isovelocity squatting are ~10% greater than concentric [12]. Taking into account that an increased eccentric load resulted in an increased eccentric knee moment and work for the knee extensors (**Fig 2**), AEL would be able to reduce the underloading that occurs during TRAD and can be recommended for inclusion in S&C practice.

In the present study, eccentric knee extensor peak moment plateaued after 120% 1RM (2.2 ± 0.3 N·m·kg^-1^). However, this value was lower than the greatest concentric moment (2.5 ± 0.5 N·m·kg^-1^ at 100% 1RM), suggesting that the underloading of the knee extensors is reduced, but not completely overcome during AEL squatting, thus we reject our third hypothesis. There are several potential reasons for this. Firstly, the relative muscular contribution of the knee extensors during a squat is ~60% compared to their single-joint isometric maximum [39], which is partially explained by the low (<50%) muscle activity during a squat compared to single-joint maximum voluntary contraction [40]. Considering that neural activation is lower during the eccentric phase compared to the concentric (**Fig 5**), the potential to produce a maximal knee extensor moment might be further reduced. These neural characteristics may therefore explain why eccentric joint moments did not exceed the concentric moments, even with AEL.

Although eccentric knee extensor moment was lower than concentric, it was hypothesised that EMG would increase as eccentric load and moment increased during AEL [38]. However, our data (**Fig 6)** showed no significant differences in vastus lateralis EMG as loads increased above 80%, despite an increase in the means for joint moment and work. This suggests factors independent of the neural input, with a plausible explanation for this being related to the force producing potential of eccentric contractions, which benefits from the spring-like behaviour of the titin myofilament [41]. Additionally, other passive-elastic tissues, such as the tendons, may also be contributing to the increased joint moments. Greater tendon forces have been observed under eccentric versus concentric conditions [42], which may be partly due to greater tendon displacement, and thus increased tendon strain [43], facilitating the storage, and subsequent release of elastic energy [44]. Therefore, we reject our fourth hypothesis that the increased eccentric joint moments during AEL are accompanied by increased muscle activation.

### Practical implications

Although the greatest peak joint moments occurred during the concentric 100% 1RM, training volume also regulates the hypertrophic stimulus [45] and greater eccentric work was performed during the AEL trials, which would facilitate a greater volume of mechanical tension. For example, comparing the eccentric work during the 120% trial to the eccentric work during a typical TRAD protocol for strength training (e.g., 80% 1RM) would result in the knee joint experiencing an increase of 37% for eccentric work, as well as a 17% increase in the eccentric peak moment. A training load that maximises the joint moment, but also facilitates multiple repetitions at a high load is likely to optimise hypertrophy and strength gains. Therefore, according to the present study using the Kineo system, it appears this may be best achieved with AEL training using a load of 120% 1RM during the eccentric phase of the squat. It remains to examine whether the kinetics during AEL squatting are altered as progressive repetitions are performed, as previous research has demonstrated a decrease in knee extension moment and a compensatory increase in hip extension moment under traditional loading to volitional failure [46].

Our data suggests that the knee extensors are preferentially loaded during eccentric squatting, experiencing greater peak moments and muscle activity than the hip extensors. Therefore, squatting with AEL may benefit sporting activities that rely heavily on the knee extensors such as cycling [47], rowing [48], and sprinting [49]. Furthermore, AEL squatting may elicit eccentric-specific adaptation in the form of an increased fascicle length [50], and thus contraction velocity. As there appears to be a preferential loading of the quadriceps, these eccentric-specific adaptations may prove beneficial to changes of direction and braking ability [51], and injury prevention/rehabilitation of the knee [52]. Using the data collected in this study, future training intervention research should test whether these loading characteristics of AEL training translate into the hypothesised improvements in performance.

## Conclusion

In conclusion, the knee extensors were preferentially loaded during eccentric squatting, and demonstrated increasing joint moment and work as eccentric load increased, with eccentric knee joint moment plateauing at 120% 1RM. However, despite eccentric contractions having the potential to produce the greatest joint moments, AEL squatting did not elicit an eccentric knee joint moment greater than the concentric joint moments produced during a one-repetition maximum. Increasing eccentric load resulted in a greater volume of work (suggesting an increased volume of mechanical tension) specifically in the earlier phase of the descent, which may in turn enhance the stimulus for hypertrophic adaptation. The data from this study suggests than an AEL of 120% 1RM should maximises knee extensor loading during the eccentric phase of the AEL squat. Future research will be needed to confirm if the greater loading results in increased training adaptations.

## Supporting information

S1 File (DOCX)

## References

[1] Weyand P.G., et al., Faster top running speeds are achieved with greater ground forces not more rapid leg movements. J Appl Physiol (1985), 2000. 89(5): p. 1991–9. 11053354 10.1152/jappl.2000.89.5.1991

[2] Barker L.A., Harry J.R., and Mercer J.A., Relationships Between Countermovement Jump Ground Reaction Forces and Jump Height, Reactive Strength Index, and Jump Time. J Strength Cond Res, 2018. 32(1): p. 248–254. doi: 10.1519/JSC.0000000000002160 28746248

[3] Folland J.P. and Williams A.G., The adaptations to strength training: morphological and neurological contributions to increased strength. Sports Med, 2007. 37(2): p. 145–68. doi: 10.2165/00007256-200737020-00004 17241104

[4] Balshaw T.G., et al., Changes in agonist neural drive, hypertrophy and pre-training strength all contribute to the individual strength gains after resistance training. European journal of applied physiology, 2017. 117(4): p. 631–640. 28239775 10.1007/s00421-017-3560-x

[5] Hartman J.W., Moore D.R., and Phillips S.M., Resistance training reduces whole-body protein turnover and improves net protein retention in untrained young males. Applied physiology, nutrition, and metabolism, 2006. 31(5): p. 557–564. 10.1139/h06-031 17111010

[6] Drummond M.J., et al., Nutritional and contractile regulation of human skeletal muscle protein synthesis and mTORC1 signaling. Journal of applied physiology, 2009. 106(4): p. 1374–1384. 19150856 10.1152/japplphysiol.91397.2008 PMC2698645

[7] Wackerhage H., et al., Stimuli and sensors that initiate skeletal muscle hypertrophy following resistance exercise. J Appl Physiol (1985), 2018(126): p. 30–43. 10.1152/japplphysiol.00685.2018 30335577

[8] Rindom E., et al., Activation of mTORC1 signalling in rat skeletal muscle is independent of the EC-coupling sequence but dependent on tension per se in a dose-response relationship. Acta Physiol (Oxf), 2019. 227(3): p. e13336. doi: 10.1111/apha.13336 31231946

[9] Kumar V., et al., Muscle protein synthetic responses to exercise: effects of age, volume, and intensity. Journals of Gerontology Series A: Biomedical Sciences and Medical Sciences, 2012. 67(11): p. 1170–1177. 10.1093/gerona/gls141 22859389

[10] Ahtiainen J.P., et al., Muscle hypertrophy, hormonal adaptations and strength development during strength training in strength-trained and untrained men. European journal of applied physiology, 2003. 89(6): p. 555–563. 12734759 10.1007/s00421-003-0833-3

[11] Suchomel T.J., et al., Implementing eccentric resistance training—Part 1: A brief review of existing methods. Journal of Functional Morphology and Kinesiology, 2019. 4(2): p. 38. 10.3390/jfmk4020038 PMC7739257 33467353

[12] Armstrong R., et al., Determining concentric and eccentric force–velocity profiles during squatting. European Journal of Applied Physiology, 2022: p. 1–11. 35038023 10.1007/s00421-021-04875-2 PMC8854263

[13] Herzog W., Why are muscles strong, and why do they require little energy in eccentric action? J Sport Health Sci, 2018. 7(3): p. 255–264. doi: 10.1016/j.jshs.2018.05.005 30356622 PMC6189244

[14] Hessel A.L., Lindstedt S.L., and Nishikawa K.C., Physiological mechanisms of eccentric contraction and its applications: a role for the giant titin protein. Frontiers in physiology, 2017. 8: p. 70. doi: 10.3389/fphys.2017.00070 28232805 PMC5299520

[15] Edman K.A., Double-hyperbolic force-velocity relation in frog muscle fibres. J Physiol, 1988. 404: p. 301–21. 3267024 10.1113/jphysiol.1988.sp017291 PMC1190827

[16] Hahn D., Stretching the limits of maximal voluntary eccentric force production in vivo. J Sport Health Sci, 2018. 7(3): p. 275–281. 30356655 10.1016/j.jshs.2018.05.003 PMC6189274

[17] Duchateau J. and Enoka R.M., Neural control of lengthening contractions. J Exp Biol, 2016. 219(Pt 2): p. 197–204. 26792331 10.1242/jeb.123158

[18] Behm D.G., Power K.E., and Drinkwater E.J., Muscle activation is enhanced with multi-and uni-articular bilateral versus unilateral contractions. Canadian journal of applied physiology, 2003. 28(1): p. 38–52. 12671194 10.1139/h03-004

[19] Swinton P.A., et al., A biomechanical comparison of the traditional squat, powerlifting squat, and box squat. J Strength Cond Res, 2012. 26(7): p. 1805–16. 22505136 10.1519/JSC.0b013e3182577067

[20] Harden M., et al., Four weeks of augmented eccentric loading using a novel leg press device improved leg strength in well-trained athletes and professional sprint track cyclists. PLoS One, 2020. 15(7): p. e0236663. doi: 10.1371/journal.pone.0236663 32726364 PMC7390385

[21] Walker S., et al., Acute elevations in serum hormones are attenuated after chronic training with traditional isoinertial but not accentuated eccentric loads in strength-trained men. Physiol Rep, 2017. 5(7). 10.14814/phy2.13241 PMC5392527 28400506

[22] Harden M., et al., Exploring the practical knowledge of eccentric resistance training in high-performance strength and conditioning practitioners. International Journal of Sports Science & Coaching, 2020. 15(1): p. 41–52.

[23] Douglas J., et al., Chronic Adaptations to Eccentric Training: A Systematic Review. Sports Med, 2017. 47(5): p. 917–941. doi: 10.1007/s40279-016-0628-4 27647157

[24] Harden M., et al., An Evaluation of Supramaximally Loaded Eccentric Leg Press Exercise. Journal of strength and conditioning research, 2018. 32(10). 10.1519/JSC.0000000000002497 29470362

[25] Sarto F., et al., Muscle activation during leg-press exercise with or without eccentric overload. Eur J Appl Physiol, 2020. 120(7): p. 1651–1656. 32447452 10.1007/s00421-020-04394-6

[26] Escamilla R.F., et al., Effects of technique variations on knee biomechanics during the squat and leg press. Med Sci Sports Exerc, 2001. 33(9): p. 1552–66. doi: 10.1097/00005768-200109000-00020 11528346

[27] Wagle J.P., et al., Accentuated Eccentric Loading and Cluster Set Configurations in the Back Squat: A Kinetic and Kinematic Analysis. J Strength Cond Res, 2021. 35(2): p. 420–427. doi: 10.1519/JSC.0000000000002677 29927889

[28] van den Tillaar R., Effect of Descent Velocity upon Muscle Activation and Performance in Two-Legged Free Weight Back Squats. Sports (Basel), 2019. 7(1). doi: 10.3390/sports7010015 30621028 PMC6359524

[29] Miletello W.M., Beam J.R., and Cooper Z.C., A biomechanical analysis of the squat between competitive collegiate, competitive high school, and novice powerlifters. The Journal of Strength & Conditioning Research, 2009. 23(5): p. 1611–1617. 19620900 10.1519/JSC.0b013e3181a3c6ef

[30] Flanagan S.P. and Salem G.J., Lower extremity joint kinetic responses to external resistance variations. J Appl Biomech, 2008. 24(1): p. 58–68. doi: 10.1123/jab.24.1.58 18309184

[31] Jeffreys I., Warm up revisited–the ‘ramp’method of optimising performance preparation. UKSCA Journal, 2006. 6: p. 15–19.

[32] Haff G.G. and Triplett N.T., Essentials of strength training and conditioning 4th edition. 2015: Human kinetics.

[33] Schwartz M.H. and Rozumalski A., A new method for estimating joint parameters from motion data. Journal of biomechanics, 2005. 38(1): p. 107–116. 15519345 10.1016/j.jbiomech.2004.03.009

[34] Hermens H.J., et al., European recommendations for surface electromyography. Roessingh research and development, 1999. 8(2): p. 13–54.

[35] Field A., Discovering Statistics Using IBM SPSS Statistics 4th edition. 4th ed. 2013: Sage Publications. 915.

[36] Farris D.J., et al., Deconstructing the power resistance relationship for squats: A joint-level analysis. Scand J Med Sci Sports, 2016. 26(7): p. 774–81. doi: 10.1111/sms.12508 26103786

[37] Kellis E., Arambatzi F., and Papadopoulos C., Effects of load on ground reaction force and lower limb kinematics during concentric squats. Journal of Sports Sciences, 2005. 23(10): p. 1045–1055. 16194981 10.1080/02640410400022094

[38] Luera M.J., Stock M.S., and Chappell A.D., Electromyographic amplitude vs. concentric and eccentric squat force relationships for monoarticular and biarticular thigh muscles. The Journal of Strength & Conditioning Research, 2014. 28(2): p. 328–338. 23897014 10.1519/JSC.0b013e3182a1f434

[39] Bryanton M.A., et al., Effect of squat depth and barbell load on relative muscular effort in squatting. J Strength Cond Res, 2012. 26(10): p. 2820–8. doi: 10.1519/JSC.0b013e31826791a7 22797000

[40] Yavuz H.U., et al., Kinematic and EMG activities during front and back squat variations in maximum loads. Journal of sports sciences, 2015. 33(10): p. 1058–1066. 25630691 10.1080/02640414.2014.984240

[41] Herzog W., The role of titin in eccentric muscle contraction. Journal of Experimental Biology, 2014. 217(16): p. 2825–2833. 25122914 10.1242/jeb.099127

[42] Finni T., et al., Comparison of force-velocity relationships of vastus lateralis muscle in isokinetic and in stretch-shortening cycle exercises. Acta Physiol Scand, 2003. 177(4): p. 483–91. 12648166 10.1046/j.1365-201X.2003.01069.x

[43] Maganaris C.N. and Paul J.P., In vivo human tendon mechanical properties. J Physiol, 1999. 521 Pt 1(Pt 1): p. 307–13. 10562354 10.1111/j.1469-7793.1999.00307.x PMC2269645

[44] Alexander R.M., Tendon elasticity and muscle function. Comp Biochem Physiol A Mol Integr Physiol, 2002. 133(4): p. 1001–11. 12485689 10.1016/s1095-6433(02)00143-5

[45] Krieger J.W., Single vs. multiple sets of resistance exercise for muscle hypertrophy: a meta-analysis. J Strength Cond Res, 2010. 24(4): p. 1150–9. doi: 10.1519/JSC.0b013e3181d4d436 20300012

[46] Brice S.M., et al., Impact of performing heavy-loaded barbell back squats to volitional failure on lower limb and lumbo-pelvis mechanics in skilled lifters. J Sports Sci, 2020. 38(1): p. 100–105. doi: 10.1080/02640414.2019.1683385 31638481

[47] Aasvold L.O., Ettema G., and Skovereng K., Joint specific power production in cycling: The effect of cadence and intensity. PLoS One, 2019. 14(2): p. e0212781. doi: 10.1371/journal.pone.0212781 30794700 PMC6386487

[48] Baudouin A. and Hawkins D., A biomechanical review of factors affecting rowing performance. British journal of sports medicine, 2002. 36(6): p. 396–402. 12453833 10.1136/bjsm.36.6.396 PMC1724573

[49] Dowson M., et al., Modelling the relationship between isokinetic muscle strength and sprint running performance. Journal of sports sciences, 1998. 16(3): p. 257–265. 9596360 10.1080/026404198366786

[50] Timmins R.G., et al., Architectural Changes of the Biceps Femoris Long Head after Concentric or Eccentric Training. Med Sci Sports Exerc, 2016. 48(3): p. 499–508. doi: 10.1249/MSS.0000000000000795 26460634

[51] Colby S., et al., Electromyographic and kinematic analysis of cutting maneuvers. Implications for anterior cruciate ligament injury. Am J Sports Med, 2000. 28(2): p. 234–40. doi: 10.1177/03635465000280021501 10751001

[52] Lorenz D. and Reiman M., The role and implementation of eccentric training in athletic rehabilitation: tendinopathy, hamstring strains, and acl reconstruction. Int J Sports Phys Ther, 2011. 6(1): p. 27–44. 21655455 PMC3105370

