# Supplementary material for: An investigation of movement dynamics and muscle activity during traditional and accentuated-eccentric squatting
Source: PLoS One. 2022 Nov 1;17(11):e0276096. doi: 10.1371/journal.pone.0276096 (PMC9624406; doi:10.1371/journal.pone.0276096)
Supplement: S1 File — (DOCX) [file pone.0276096.s001.docx]

**An investigation of movement dynamics and muscle activity during traditional and accentuated-eccentric squatting**

**Supplementary material, providing full details of:**

A comparison of kinematics and muscle activity between squats performed with barbells and the Kineo training system

Richard Armstrong^1*¶^; Vasilios Baltzopoulos^1¶^; Carl Langan-Evans^1¶^; Dave Clark^1¶^; Jonathan Jarvis^1¶^; Claire Stewart^1¶^; & Thomas O’Brien^1¶^.

^1^Research Institute for Sport and Exercise Science, Liverpool John Moores University, Liverpool, United Kingdom

* Corresponding Author

E-Mail: [R.Armstrong@2014.LJMU.ac.uk](mailto:R.Armstrong@2014.LJMU.ac.uk) (RA)

¶ These authors contributed equally to this work

**Introduction**

Recently, digital controllers have been combined with resistance training apparatus in order to manipulate how the external load is applied during resistance training, to generate ‘Smart Training Systems’ [1]. One commercially available unit is the Kineo Training System (V7.0, GLOBUS, Italy), which connects a computer-controlled motorised cable-pulley system to a shoulder/hip harness, facilitating a variety of open and closed chain exercises including the Kineo squat. No previous work has directly compared the kinematics or muscle activity between the Kineo squat and the barbell back squat or barbell front squat. Although previous research has compared barbell squatting to the belt-squat [2, 3], the Kineo uses a shoulder/hip harness, rather than a hip-only belt, and thus data from the previous belt-squat studies may not be transferrable to the Kineo squat.

Therefore, the aim of these measurements were 1) to investigate how the squatting variation affects the kinematics of the hip, knee, and ankle joints. 2) to investigate whether these changes in kinematics are accompanied by changes in activity of the vastus lateralis and gluteus maximus.

**Materials and methods**

**Participants**

Twelve resistance trained males (age: 25 ± 2 years, mass: 78 ± 7 kg, height: 179 ± 6 cm) were recruited. All participants had a minimum of 2 years resistance training experience, and were proficient in the squatting exercise, as assessed by a qualified strength and conditioning coach according to the criteria in ‘familiarisation’ below. All participants provided written informed consent before the start of data collection and the Liverpool John Moores University research ethics committee approved the study (UREC code: 21/SPS/035).

**Experimental protocol**

Participants reported to the Liverpool John Moores University laboratories on three occasions. The first two visits were used for familiarisation with the Kineo on which the Kineo squat was performed, and to ensure all participants had proficient squatting technique in all three squatting variations. Experimental data were collected on the third day, at four to seven days after the familiarisation on day two. During this visit, body mass and height of the participant were collected, along with kinematics of the lower limbs and electromyography (EMG) of the vastus lateralis and gluteus maximus during the three squatting variations.

**Familiarisation**

In two familiarisation sessions, participants were introduced to the Kineo Training System on which they would perform the Kineo squat. The Kineo uses a shoulder/hip harness to load the participant, which is then attached via a cable to a powerful servomotor. This results in slight anterior loading, similar to that seen during the front squat. Following a warm-up with the RAMP protocol [4], participants performed each of the three squatting variations with increasing external loads up to 100% of body mass. During each squatting variation, technique was assessed for proficiency by a qualified strength and conditioning coach to ensure a range of motion that allows the centre of the hip joint to finish below the centre of the knee joint (parallel squat), maintenance of a full foot-floor contact, maintenance of normal spinal curvature, and tracking of the knees in line with the feet.

**Kinematic and electromyography testing**

Participants reported to the laboratory for the experimental day having refrained from strenuous physical activity for 72 hours. Participant body mass (kg) and height (cm) were collected (SECA 704/202, Germany), the participants then completed the standardised warmup and were fitted with surface EMG electrodes (BlueSensor, Ambu, Denmark) and reflective markers. Before electrode placement, the skin over the vastus lateralis and gluteus maximus was shaved, abraded, and cleaned with an isopropyl alcohol swab (70%) to improve signal clarity. EMG electrodes were positioned following the recommendations of the SENIAM project [5]. To allow normalisation of EMG signals, participants performed a maximal isometric voluntary contraction for the knee extensors in a seated position with the knee flexed at 80°, and maximal isometric voluntary contraction of the hip extensors in a prone position with the hip flexed at 30° (0° represent full extension for both hip and knee joints). EMG signals from experimental trials were normalised against these isometric values.

A 36-marker set utilising technical and anatomical markers were used to track kinematics of the pelvis and lower limbs. This included a modified CODA pelvis marker set (additional tracking markers located on the iliac crest) to account for ASIS occlusion during hip flexion. The remaining markers tracked the thigh, shank, and feet segments (lateral & medial femoral epicondyles, lateral & medial malleoli, heel, 1^st^ & 5^th^ metatarsals, thigh cluster, & shank cluster). Functional joint analyses were performed to calculate the hip and knee joint centres utilising the Gilette algorithm [6].

Pelvic tilt angle was determined with respect to the global coordinate system [7], with a positive angle representing anterior pelvic tilt, and negative angle representing posterior pelvic tilt about the mediolateral axis. This results in an anterior pelvic tilt angle of ~10° when standing upright [8]. Hip angle was determined from the thigh segment in relation to the pelvis rotating about the determined functional hip joint centre. Knee angle was determined from the shank segment in relation to the thigh segment rotating about the determined functional knee joint axis, with an angle of 0° representing full hip and knee extension, respectively. Ankle angle was determined from the foot segment in relation to the shank segment, rotating about the mediolateral axis. An ankle angle of 0° represents a neutral ankle position when standing upright, with a positive joint ankle representing ankle dorsiflexion.

Participants performed each squatting variation in a randomised order. For each variation, three repetitions were performed at 50%, 85%, and 100% of body mass. Each trial was separated by 5 minutes passive recovery.

**Data acquisition and analyses**

Electromyography signals were wirelessly transmitted (Research DTS, Noraxon, USA) (sampling at 1500 Hz) to a desktop computer. A six, 3D-motion capture camera system (Opus 3 series, Qualisys, Sweden) (sampling at 200 Hz), was used to track the reflective markers. Motion and EMG data were collected synchronously in Qualisys Track Manager (Qualisys, Sweden) and then exported to Visual 3D (C-Motion, USA) to undergo analyses. Motion data were lowpass filtered (4^th^ order Butterworth) with a 6 Hz cut-off frequency. EMG data were processed via a 10-250 Hz band pass filter, before a root mean squared moving average of 100 ms. Motion data allowed for quantification of peak joint angle (°), range of motion (°), and joint velocity (°·s^-1^), and the electromyography data allowed for quantification of muscle activity, normalised to isometric maximum (%).

**Statistical analyses**

All statistical analyses were performed in SPSS (v27, IBM, USA), with statistical significance determined by an alpha level of 0.05. A two-way repeated measures ANOVA, with Bonferroni post-hoc analysis (squat variation x squat load) was used to assess whether the squat variation and/or load influenced squatting kinematics/muscle activity. All data is reported as mean ± SD. Effect sizes were calculated for all ANOVA’s that displayed significance tests using ω^2^, with values of 0.01, 0.06, and 0.14 indicating a small, medium and large effect size, respectively [9]. Coefficient of variation (%) was used to identify intra-trial reliability.

**Results**

Analyses of joint ranges of motion showed that there was no significant effect of squatting variation on the range of motion for the hip (76 ± 9°) (F = 0.338, *P* = 0.719), knee (123 ± 9°) (F = 3.365, *P* = 0.109), or ankle joints (35 ± 3°) (F = 1.295, *P* = 0.281). However, there was a medium effect of squatting variation on pelvis range of motion (F = 4.127, *P* = 0.039, ω^2^ = 0.08), with the Kineo squat (11 ± 8°) having a significantly smaller pelvic range of motion than both the barbell back squat (21 ± 6°) and barbell front squat (20 ± 5°) (**Fig 1)**. External load had no effect on joint range of motion (*P* = 0.090-0.754). Therefore, all subsequent discussion of joint ranges of motion refers to the 100% trial.


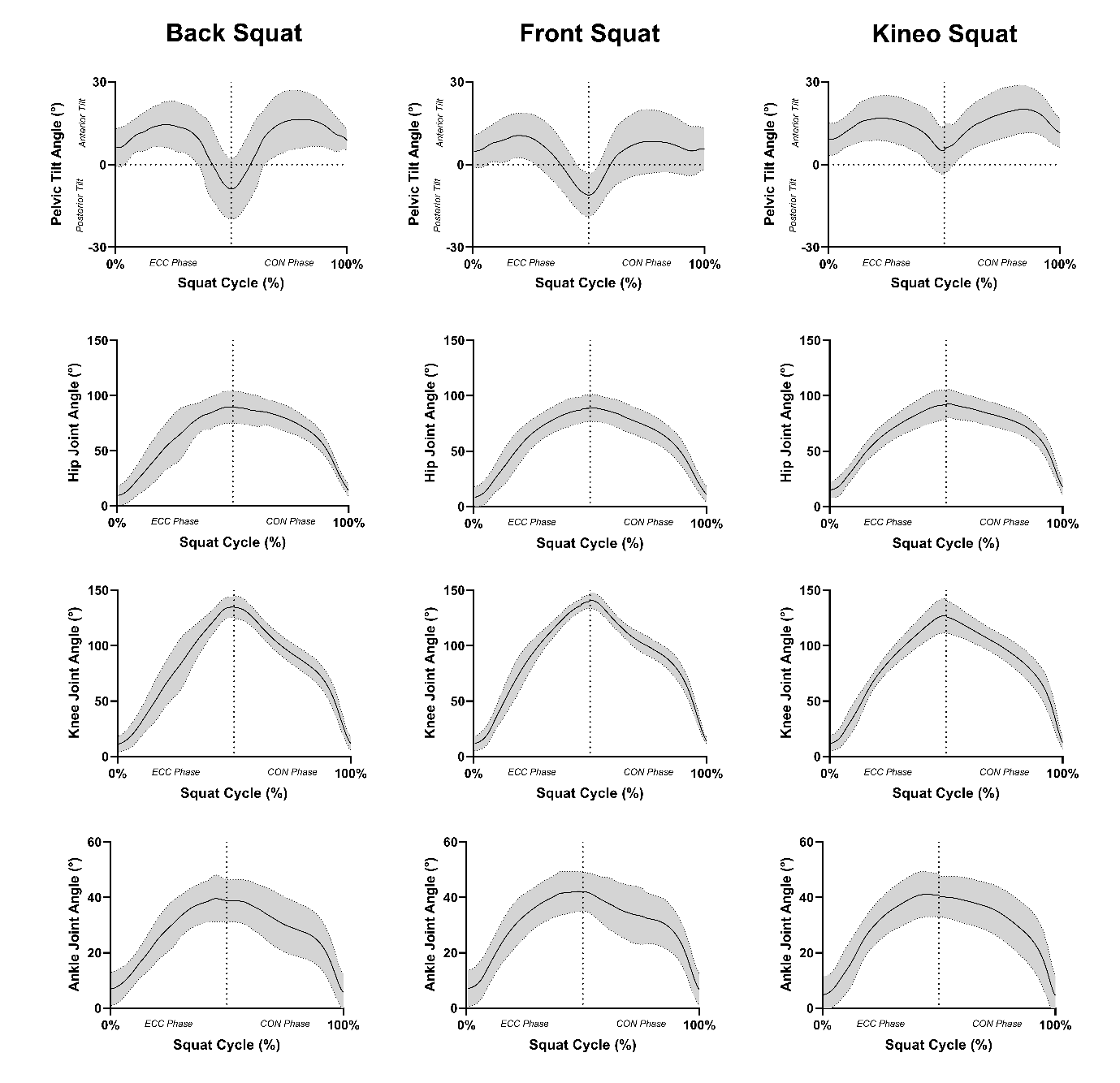


**Fig 1.** Mean ± SD joint angle (°) during the eccentric phase and concentric phase of the barbell back squat, barbell front squat, and Kineo squat with an external load of 100% of body mass. Positive pelvic tilt angle is representative of anterior pelvic tilt, with a negative angle being representative of posterior pelvic tilt. Positive ankle angle is representative of dorsiflexion, with a negative angle being representative of plantar flexion.

Analyses of the angular joint velocities showed no effect of squatting variation on the hip joint velocity (F = 0.712, *P* = 0.508) **(Fig 2).** However, there was a large effect of squatting variation on knee joint velocity (F = 12.121, *P* <0.001, ω^2^ = 0.23) (**Fig 3**), with the Kineo squat displaying significantly greater knee joint velocity than the barbell back squat (*P* = 0.008) and barbell front squat (*P* = 0.005), with no difference found between the barbell back squat and barbell front squat (*P* = 0.701). However, this only occurred under the 50% loading condition, no significant differences were found between the 3 squat variations at the higher loads (*P* > 0.05).


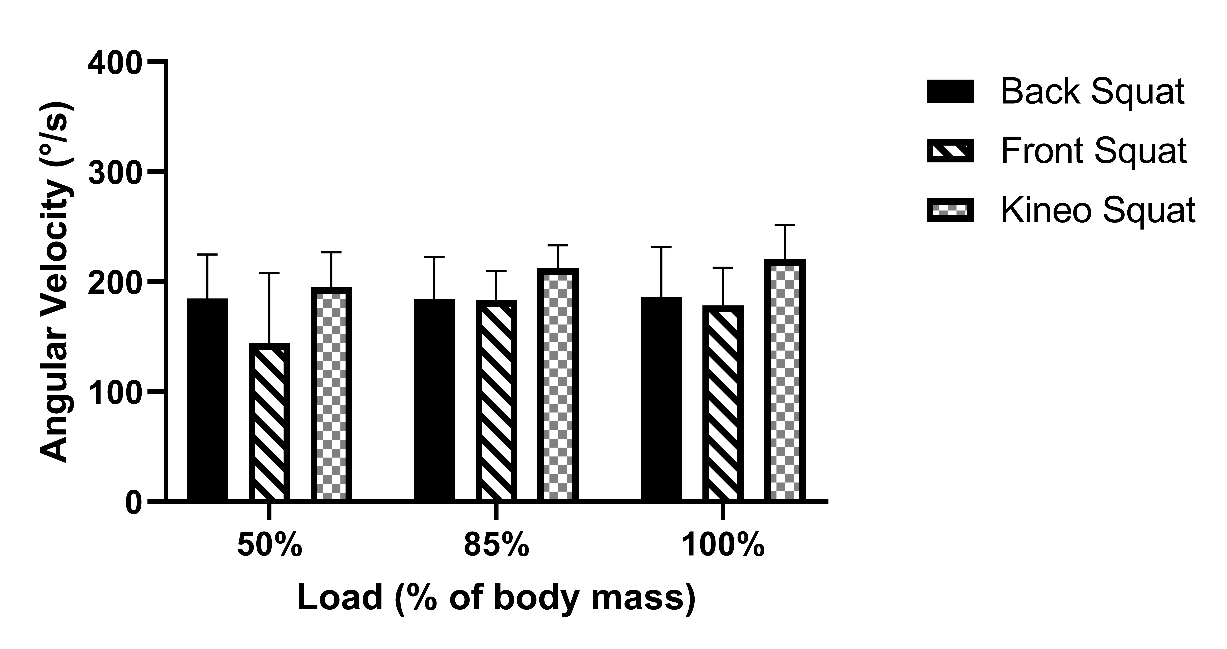


**Fig 2.** Mean ± SD hip joint angular velocity (°/s) during the concentric phase of the barbell back squat, barbell front squat, and Kineo squat with an external load of 50. 85, & 100% of body mass.


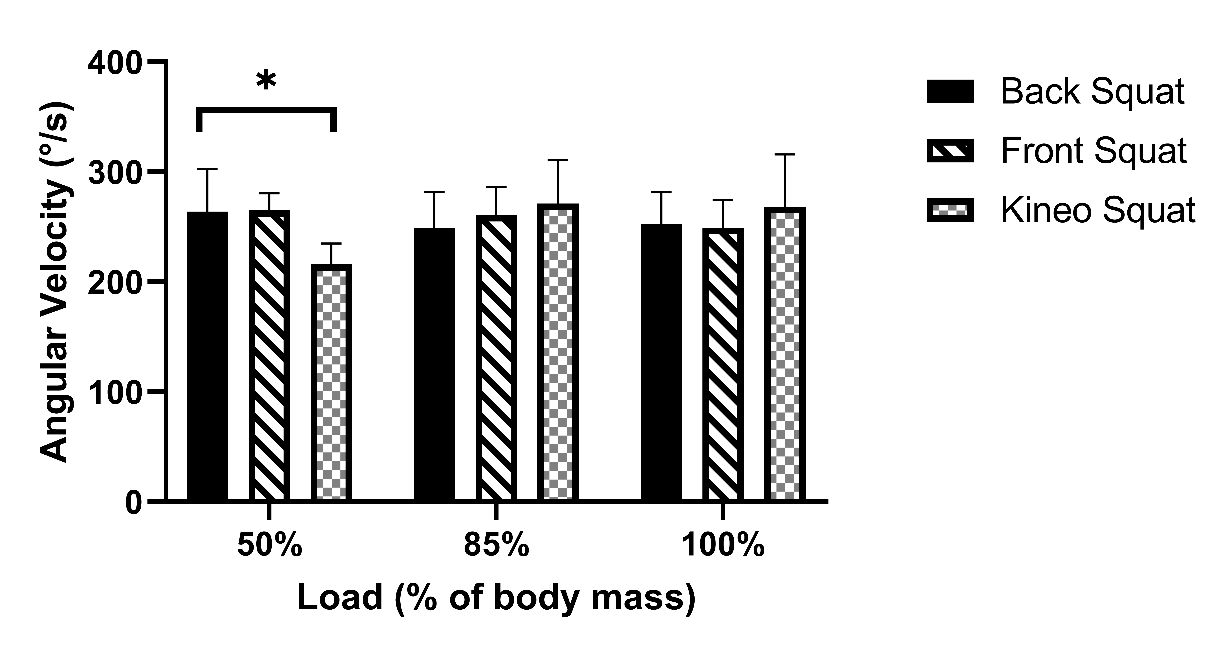


**Fig 3.** Mean ± SD knee joint angular velocity (°/s) during the concentric phase of the barbell back squat, barbell front squat, and Kineo squat with an external load of 50, 85, & 100% of body mass.

Analyses of muscle activity showed no effect of squat variation on gluteus maximus activity (F = 1.79, *P* = 0.203) (**Fig 4**). However, there was a medium effect of squat variation on vastus lateralis activity (F = 4.445, *P* = 0.032, ω^2^ = 0.08) **(Fig 5**)**,** with the barbell front squat and Kineo squat having a significantly greater activity than the barbell back squat (*P* = 0.022). There was a large effect of loading on both the gluteus maximus (F = 40.271, *P* <0.001, ω^2^ = 0.53) and vastus lateralis activity (F = 24.69, *P* <0.001, ω^2^ = 0.49). Muscle activity increased as external load increased.


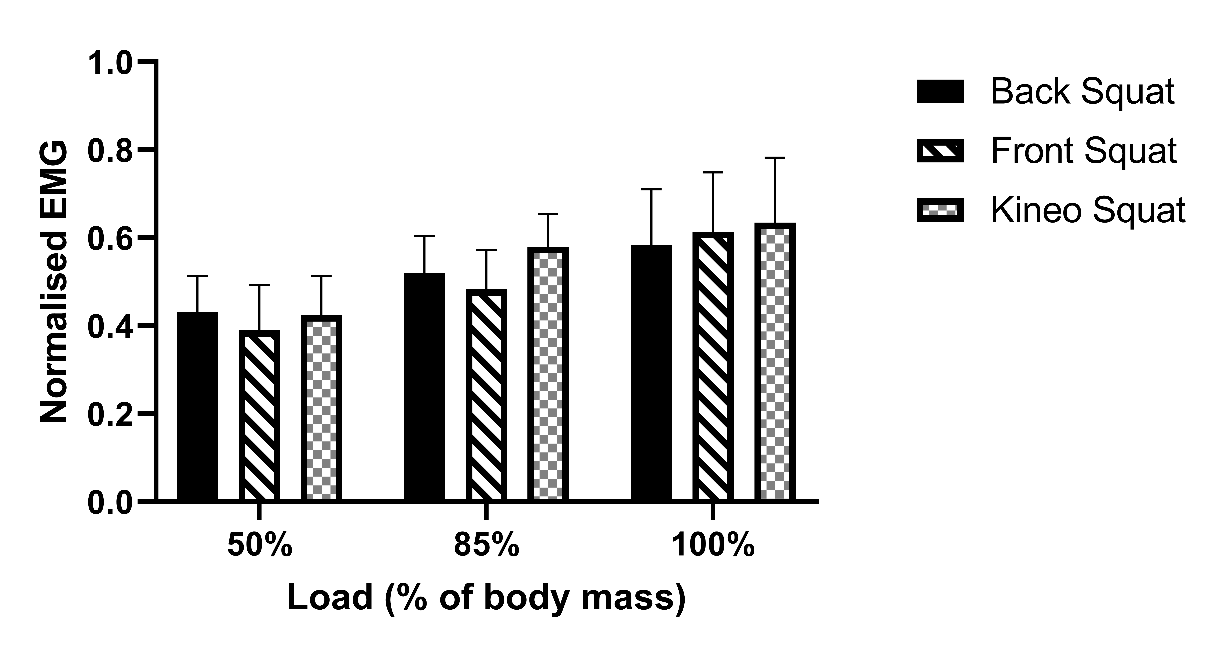


**Fig 4.** Mean ± SD gluteus maximus normalised EMG during the concentric phase of the barbell back squat, barbell front squat, and Kineo squat with an external load of 50, 85, & 100% of body mass.


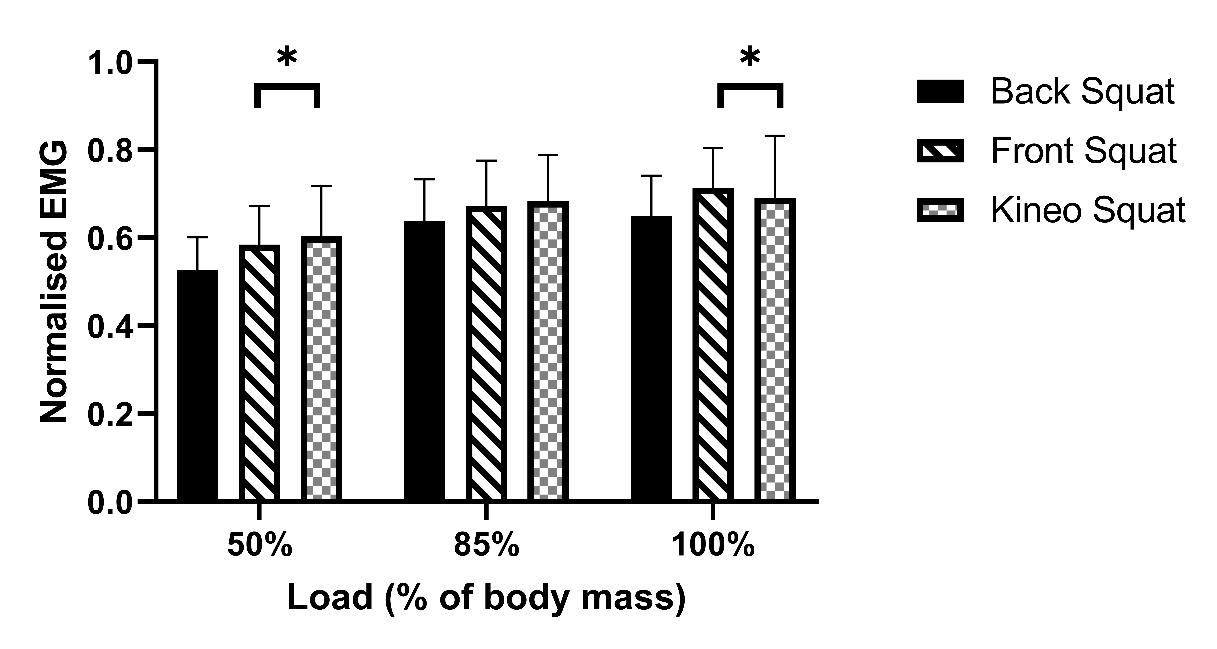


**Fig 5.** Mean ± SD vastus lateralis normalised EMG during the concentric phase of the barbell back squat, barbell front squat, and Kineo squat with an external load of 50, 85, & 100% of body mass. * = significantly greater EMG than during the barbell back squat.

During the concentric phase of the squat, the peak vastus lateralis activity was at a significantly greater knee flexion angle for the barbell back squat (119 ± 13°) and barbell front squat (121 ± 11°) than the Kineo squat (105 ± 17°) (F = 11.286, *P* <0.001, ω^2^ = 0.06). There was no effect of squat variation of the hip joint angle at which peak gluteus maximus activity occurred (51 ± 5°) (F = 3.622, *P* = 0.092). There was also no effect of loading of the joint angle at which the peak vastus lateralis activity (F = 0.08, *P* = 0.923) or gluteus maximus activity occurred (F = 0.281, *P* = 0.759).

Analyses of intra-trial reliability revealed coefficients of variations for the hip, knee, ankle, and pelvis ranges of motion to be 2%, 1.5%, 1.3%, and 10.6%, respectively. CV for hip and knee peak joint velocities were 7.8% and 6.7%, respectively and for gluteus maximus and vastus lateralis EMG 17.3% and 6.6%, respectively. Finally, the CV for the hip and knee joint angles at which peak EMG activity occurred for the gluteus maximus and vastus lateralis to be 10.8% and 7.3%, respectively.

**Interpretation and Conclusion**

There has been ongoing debate about whether there are differences in lower limb kinematics and muscle activity between squat variations. The present findings suggest that there are no differences in hip, knee, and ankle joint ranges of motion between squat variations in resistance trained individuals, and that external load up to 100% body mass has no influence on these ranges of motion. However, there is greater vastus lateralis muscle activity in squat variations that are loaded anteriorly (i.e. barbell front squat and Kineo squat) than in posteriorly loaded variations (i.e. barbell back squat). Regardless of variation, no differences were found in gluteus maximus muscle activity. All squat variations produce a sufficient muscle activity to promote adaptations to the hip and knee extensors, and the greatest vastus lateralis activity is during the first 10% of the concentric phase. Our results also show that the Kineo squat may reduce posterior pelvic tilt during the transition from the eccentric to concentric phase of the squat, which may reduce lower back shear and compression forces.

**References**

1. West, A., J. Smith, and C. McLeod, *Development and initial evaluation of a smart resistance training system.* Proceedings of the Institution of Mechanical Engineers, Part P: Journal of Sports Engineering and Technology, 2009. **223**(1): p. 31-47.

2. Joseph, L., et al., *Activity of Trunk and Lower Extremity Musculature: Comparison Between Parallel Back Squats and Belt Squats.* J Hum Kinet, 2020. **72**: p. 223-228.

3. Evans, T.W., et al., *Comparison of Muscle Activation Between Back Squats and Belt Squats.* J Strength Cond Res, 2019. **33 Suppl 1**: p. S52-s59.

4. Jeffreys, I., *Warm up revisited–the ‘ramp’method of optimising performance preparation.* UKSCA Journal, 2006. **6**: p. 15-19.

5. Hermens, H.J., et al., *European recommendations for surface electromyography.* Roessingh research and development, 1999. **8**(2): p. 13-54.

6. Schwartz, M.H. and A. Rozumalski, *A new method for estimating joint parameters from motion data.* Journal of biomechanics, 2005. **38**(1): p. 107-116.

7. Lewis, C.L., et al., *Differences in Lower Extremity and Trunk Kinematics between Single Leg Squat and Step Down Tasks.* PLoS One, 2015. **10**(5): p. e0126258.

8. Lewis, C.L., et al., *The Human Pelvis: Variation in Structure and Function During Gait.* Anat Rec (Hoboken), 2017. **300**(4): p. 633-642.

9. Field, A., *Discovering Statistics Using IBM SPSS Statistics*. 4th ed. 2013: Sage Publications. 915.
